# Habitat-driven ecological strategies shape Chinese pine functional traits and rhizosphere metabolites in Qinling Mountains, China

**DOI:** 10.3389/fpls.2025.1690544

**Published:** 2025-11-18

**Authors:** Hang Yang, Yue Pang, Haibin Kang, Yahui Song, Kunlin Hu, Yuchao Wang, Dexiang Wang

**Affiliations:** 1 College of Forestry, Northwest A&F University, Yangling, Shaanxi, China; 2 College of Forestry, Hebei Agricultural University, Baoding, China; 3 Shangluo Qinling Ecological Protection Office, Shangluo, China; 4 Xi’an Botanical Garden of Shaanxi Province, Xi’an, China

**Keywords:** plant functional traits, rhizosphere soil metabolites, ecological adaptation strategies, different habitats, *P.tabuliformis*

## Abstract

**Aims:**

Plant functional traits (PFTs) and rhizosphere soil metabolites (RSMs) play crucial roles in the connection between plants and soil environments, influencing plant ecological adaptation processes. However, the strategies and driving factors underlying their variation in different habitats are poorly understood.

**Methods:**

In this study, we investigated *P.tabuliformis* population traits and RSMs in two habitats, assessed trait and metabolite variations, and analyzed how traits and metabolites were associated with plant strategies.

**Results:**

The first principal component analysis (PCA) axis of the PFTs reflected trade-offs in traits linked to habitat: ridge habitat Chinese pine populations had high leaf dry matter content (LDMC) and fine root tissue density (FRTD) trait values that increased resource conservation, and slope habitat populations had high specific leaf area (SLA) and fine root specific root length (FRSRL) values that improved the efficiency of resource acquisition. The results of the metabolic pathway analysis of differential metabolites showed that in ridge habitats, adversity resistance-related metabolic pathways were significantly upregulated. Random forest model (RFM) analysis showed that the first PCA axis of PFTs and differential metabolites was significantly related to soil nitrogen and phosphorus content, indicating that soil nutrients are the primary factors driving the variation in *P.tabuliformis* population ecological adaptation strategies in different habitats.

**Conclusions:**

Thus, the trade-offs between PFTs and the regulation of rhizosphere soil metabolism shape the population distribution of *P.tabuliformis* in different habitats. Along the terrain gradient, soil nutrients are the primary factors driving trait and metabolite regulation-based strategies.

## 1 Introduction

Human activity is changing global environmental conditions (Matthews and Wynes, 2022), which poses significant challenges to the persistence of long-lived plant species. The persistence of the species depends on their speed of adaptation to the environment or their ability to spread to a suitable habitat (Aitken et al., 2008). However, the distribution characteristics of most plant species are a result of long-term adaptation to environmental factors, and the rates of evolution and migration of many species lag behind the pace of climate change (de Lafontaine et al., 2018; Etterson and Shaw, 2001), indicating that species must adjust their ecological survival strategies to adapt to changing environments (Weiner, 2004). Thus, understanding the potential variation rules of plant ecological adaptation strategies is of great significance for predicting how species distribution and diversity may shift with environmental changes and for determining species protection priorities. Plant functional traits (PFTs) and metabolic characteristics are key to understanding plant adaptation to environmental conditions.

It has been demonstrated that PFTs exhibit wide intraspecific and interspecific variations and are closely correlated with specific local environmental conditions (Akram et al., 2023; Zheng et al., 2019). According to plant science and optimal partitioning theory, tree species allocate resources reasonably between organs to achieve more effective resource acquisition and, therefore, maximize their performance. Thus, the ecological adaptation strategies of plants in different habitats are partly the result of limited resource allocation (Bloom et al., 1985; Hilty et al., 2021). Therefore, PFT variations and trade-offs characterize plant adaptation strategies to different environmental filters or interferences (Westoby et al., 2002; Wright et al., 2007). Previous studies have found extensive intraspecific and interspecific trait variations along climatic and geographical gradients (Fonseca et al., 2000; Niinemets, 2001; Ordonez et al., 2009). By studying variations on a global scale, ecologists have revealed basic strategies for plant survival and evolution in different environments (Bisbing et al., 2020; Díaz, 2016; Kelly et al., 2021). The most widely accepted trait-based fundamental strategy axis in plants is from resource conservation to acquisition (Díaz et al., 2004; Reich et al., 1998). Plants at the end of the conservative strategy axis generally have relatively smaller and specific leaf areas, higher leaf carbon content, and lower leaf nitrogen content, whereas plants with resource-acquisition strategies at the other end of the axis have the opposite traits. Plants growing in comfortable or resource-rich environments typically adopt resource-acquisitive strategies to increase the efficiency of competition for resources, whereas the opposite is true for plants that grow in harsh environmental conditions.

Plant rhizosphere soil metabolites (RSMs), which are natural products of long-term mutual feedback between plants and soil, are gaining momentum as measurable and informative functional root traits (Jain et al., 2019; Williams et al., 2022). RSMs are intrinsically linked to PFTs, as both are shaped by the plant’s underlying resource use and growth strategy (Wen et al., 2022). Under this framework, conservative strategists are predicted to allocate more carbon to defense-related and recalcitrant secondary metabolites-such as phenols and terpenoids-to enhance stress tolerance and tissue protection (Huan et al., 2023). In contrast, acquisitive strategists tend to invest in metabolites that support rapid nutrient acquisition, including organic acids involved in phosphorus solubilization (Weinhold et al., 2022; Williams et al., 2022). In addition, the chemical composition and metabolic pathways of metabolites, independent of the exudation rate, affect plant growth and adaptation. Exuded metabolites may have a strong effect on plant nutrient acquisition and abiotic stress resistance by filtering or shaping the rhizosphere microbiome (van Dam and Bouwmeester, 2016; Pérez-Jaramillo et al., 2016). In addition, some exuded metabolites (organic acid metabolites) can accelerate the weathering rate of parent materials and help plants obtain more restricted nutrients (Subbarao et al., 2009). Phenolic and flavonoid metabolites are associated with plant resistance to biotic and abiotic stresses, such as UV-B radiation and drought (Huan et al., 2023; Macabuhay et al., 2021). In addition, plant RSMs and trait variations, as a response of plants to natural stresses, do not occur independently and are intricately linked (Wen et al., 2022). However, the co-change of plant traits and RSMs and the link between plant trait strategies and rhizosphere metabolites under environmental stress remain poorly understood.

In the current study, *P.tabuliformis* populations from ridge and slope habitats were selected for analysis. Variations and trade-offs in PFTs and RSM characteristics were investigated to explore the ecological adaptation strategies of *P.tabuliformis* to different habitats. Specifically, we hypothesized that (a) the PFTs and RSMs of different habitats would be affected by the conditions to which the species are adapted. (b) RSM composition is related to plant resource utilization strategies. In ridge habitats, stress-resistance-related metabolite content was negatively correlated with resource-acquisition traits and positively correlated with resource-conservation traits, indicating that plants adopt more conservative strategies.

## 2 Materials and methods

### 2.1 Study area description

This study was conducted in the Huoditang forest region in the central Qinling Mountains of Shaanxi Province, China (NSTEC: 108°21’E, 33°18’ N–108°29’E, 33°28’N). The climate is transitional between northern subtropical and warm temperate, with mean annual temperature and precipitation ranging from 8 to 10 °C and 1000 to 1200 mm, respectively. In addition, 70% of the precipitation occurs between June and September of each year. The dominant soil type was brown forest soil, with an average depth of 50 cm and a pH of 6.5. The vegetation in the study area is dominated by mixed temperate coniferous and broadleaf forests and frigid coniferous forests, and the percentage of forest cover is approximately 93.8%. The dominant tree species are *Quercus aliena* var. *acuteserrata*, *P.tabuliformis*, *P.armandii*, *Tsuga chinensis* Currently, 95% of the forest is a secondary forest that was restored after heavy felling during the 1960s (Chai and Wang, 2015; Kang et al., 2017).

### 2.2 Experimental design and sampling

To understand how trait- and RSM-based ecological adaptation strategies vary in Chinese pine in different environments, a total of 32 plots were established within the distribution range: 16 in ridge habitats 16 in slope habitats. To ensure that traits were fully expressed and to reduce ontogenetic variation, 3–5 healthy-looking adult *P. tabuliformis* individuals with similar diameters at breast height were selected as standard trees in each plot, using the mean leaf and root traits calculated from those individuals to represent the population-level traits. For leaf traits, we collected four middle-canopy branches from the east, south, west, and north of a standard tree, and current-year leaves were sampled from each branch. To assess root traits, sufficient living fine roots (< 2 mm) were collected from each standard tree in the study. Rhizosphere soil was collected using a sterile soft brush from the fine roots of 16 plots with closely matched elevations, comprising 8 plots from ridge habitats and 8 from slope habitats. One composite rhizosphere soil sample was collected and analyzed per plot. Specifically, fine root systems were carefully excavated from each plot, placed in sterile bags, and immediately transported to the laboratory on ice. In the lab, the remaining tightly adhering soil, defined as the rhizosphere soil, was then collected from the root surfaces using a sterile soft brush. After litter removal (stones, plant, and animal residues), the rhizosphere soil was frozen and stored at −80°C for metabolite determination.

For bulk soil sampling, five replicate points were selected at the four corners and center of the plot. After removing the litter layer, five soil samples (0–20 cm depth) were collected from each point using a 5 cm diameter stainless-steel auger and then fully homogenized to provide one composite sample per plot for analysis. A total of 32 soil samples were collected. All soil samples were air-dried and stored at room temperature for physicochemical analysis after sieving through a < 2 mm mesh. The coordinates and elevations of the plots were recorded using a GPSmap 60CSx (Garmin Ltd.) to mark their positions in the distribution areas.

### 2.3 Soil properties, PFT and RSM measurements

#### 2.3.1 Soil properties

Soil properties were measured following the method described by Bao (Bao, 2000). Soil pH was determined using a pH meter after shaking a soil–distilled water (1:2.5, w/v) suspension for 30 min at 200 rpm. The soil organic carbon (SOC) content of the samples was measured using the K_2_Cr_2_O_7_ oxidation method. Soil total nitrogen content was determined using a semi-automatic Kjeldahl apparatus after digestion with K_2_SO_4_:CuSO_4_·5H_2_O (10:1w/w)-H_2_SO_4_, and soil total phosphate (TP) content was determined by colorimetry using a UV spectrophotometer after digestion with HClO_4_-H_2_SO_4_. Soil nitrate nitrogen (NO_3_^–^N) and ammonium nitrogen (NH_4_^+^-N) contents were determined using a continuous flow analyzer (AA3; Germany) after extraction with 1 M KCl solution.

#### 2.3.2 Leaf traits

The needle leaf area (LA), specific leaf area (SLA), leaf dry matter content (LDMC), leaf chlorophyll (Chl a and Chl b), leaf malondialdehyde (MDA), soluble sugar content, and leaf nutrients were measured. Five fully developed and healthy acerose leaves were sampled from each branch in the current year. Leaf thickness and width were measured using a micrometer, and leaf length (LL) and width (LW) were measured using a ruler. The cambered area of a single needle leaf (S) was calculated as $S=\frac{\left(\pi \times LW\times LL\right)}{2}$, and LA was calculated as LA=2S+2LW×LL (Zhang et al., 2017). SLA was calculated as LA divided by leaf dry mass. Sufficient leaves from each branch were fully homogenized and divided into two parts. One part was cooled rapidly with liquid nitrogen and returned to the laboratory to determine the Chl, MDA, and soluble sugar contents. The other part was dried to a constant weight at 60°C after drying for 15 min at 105°C to obtain the leaf dry matter and nutrient concentrations. Chlorophyll content was measured using UV spectrophotometry after dimethyl sulfoxide extraction. MDA and soluble sugar contents were measured using UV spectrophotometry after thiobarbituric acid extraction. Leaf carbon content (LCC) was measured using the K_2_Cr_2_O_7_ oxidation method. Leaf nitrogen content (LNC) was measured using distillation titration after H_2_SO4-H_2_O_2_ digestion. Leaf phosphorus content (LPC) was determined using the vanadium molybdate yellow colorimetric method.

#### 2.3.3 Root traits

Only fine root functional traits were considered. After washing and removing surface water, the specific root length (SRL), root tissue density (RTD), and root nutrients were measured. Root diameter was measured using a micrometer, and root length was measured using a scale. Fresh weight and volume were measured using a balance and volumetric cylinder, respectively, and the root sections were dried for 48 h at 60 °C to obtain the dry mass. SRL was calculated as root length divided by root dry mass, and RTD was calculated as root dry mass divided by fresh volume (Simpson et al., 2016). The methods used to determine root carbon (RCC), nitrogen (RNC), and phosphorus content (RPC) were the same as those used for the leaves.

#### 2.3.4 RSMs

Kong’s method (Kong et al., 2022) was adopted with some modifications. Briefly, RSM extraction, UHPLC-MS/MS analysis, raw data collection, and metabolite identification were performed. (1) RSM extraction: a 50 mg soil sample was added to a 2 mL centrifuge tube with a 6 mm diameter grinding bead. A 400 μL extraction solution (methanol: water = 4:1 (v:v)) containing 0.02 mg/mL of internal standard (L-2-chlorophenylalanine) was used for metabolite extraction. Samples were ground using a Wonbio-96c frozen tissue grinder (Shanghai Wanbo Biotechnology Co., Ltd.) for 6 min (−10°C, 50 Hz), followed by low-temperature ultrasonic extraction for 30 min (5°C, 40 kHz). The samples were left at −20°C for 30 min, centrifuged for 15 min (4°C, 13000 g), and the supernatant was transferred to the injection vial for LC-MS/MS analysis. In addition, quality control samples were prepared by mixing 20 μL of the supernatants from all samples to monitor the stability of the analyses. (2) UHPLC-MS/MS analysis: LC-MS/MS sample analysis was conducted using a Thermo UHPLC-Q Exactive HF-X system equipped with an ACQUITY HSS T3 column (100 × 2.1 mm i.d., 1.8 μm, Waters, USA). The mobile phase comprised 0.1% formic acid in water:acetonitrile (95:5, v/v) (solvent A) and 0.1% formic acid in acetonitrile:isopropanol:water (47.5:47.5, v/v) (solvent B). The flow rate was 0.40 mL/min, and the column temperature was 40°C. Mass spectrometric data were collected using a Thermo UHPLC-Q Exactive HF-X mass spectrometer equipped with an electrospray ionization source operating in the positive and negative modes. The optimal conditions were as follows: source temperature at 425°C; sheath gas flow rate at 50 arb; aux gas flow rate at 13 arb; ion-spray voltage floating at −3500 V in negative mode and 3500 V in positive mode, respectively; normalized collision energy, 20–40–60 V rolling for MS/MS. The full MS resolution was 60000 and the MS/MS resolution was 7500. Data were acquired using a data-dependent acquisition mode. Detection was performed over a mass range of 70–1050 m/z (mass-to-charge ratio). (3) Raw data analysis and metabolite identification: Raw LC/MS data were pretreated using Progenesis QI (Waters Corporation, Milford, USA) software. Internal standard peaks and any known false-positive peaks (including noise, column bleed, and derivatized reagent peaks) were removed from the data matrices. Simultaneously, the metabolites were identified using HMDB (http://www.hmdb.ca/), Metlin (https://metlin.scripps.edu/), and the Majorbio database.

### 2.4 Data analysis

The mean trait value represents the average population value of a given trait. Analysis of variance (ANOVA) was used to analyze the differences in environmental factors and plant traits between different habitats after checking the normality and homogeneity of the variance of all the data. Statistical significance was set at a 0.95 confidence level (*P* < 0.05). Principal component analysis (PCA) was used to analyze the trade-offs of traits in different habitats and to screen key traits, and to identify the dominant resource-use strategies (acquisition vs. conservation) and to objectively assign each habitat’s phenotype to a strategic position in the trait space. A random forest model (RFM) was used to rank the primary influencing factors of the primary strategy axes. Pearson’s correlation analysis was used to analyze the correlations between traits and environmental factors.

The R package “ropls” (Version 1.6.2) was used to perform PCA orthogonal partial least squares discriminant analysis (OPLS-DA), and seven-cycle interactive validation to evaluate model stability. The metabolites with variable importance in projection (VIP) >1 and *P* < 0.05 were determined to be significantly different based on the VIP obtained by the OPLS-DA model and the p-value generated by Student’s t-test.

The analysis of metabolite data followed a sequential workflow to elucidate functional differences. First, differentially abundant metabolites (DAMs) between slope and ridge habitats were identified based on significant differences in relative abundance. These DAMs were then subjected to metabolic pathway enrichment analysis using the KEGG database (http://www.genome.jp/kegg/). Significantly enriched pathways were screened based on their p-values. Finally, to interpret the biological relevance, metabolites within these key pathways were analyzed for their putative functions, thereby linking metabolite patterns to potential ecological functions and revealing the metabolic basis of adaptation. Furthermore, Pearson correlation analysis was employed to examine the relationships between these metabolites and both plant functional traits and environmental factors. This analysis helped to clarify that the PFT-based PCA primarily reveals the morphological and biophysical strategy of the populations. The Python package “scipy.stats” (https://docs.scipy.org/doc/scipy/) was used to perform enrichment analysis to obtain the most relevant biological pathways for the experimental treatments. RFM was used to rank the primary factors influencing the differential metabolites. All data were sorted using Microsoft Excel 2019 software. PCA, RFM, and analysis of differential metabolites were conducted using R 4.03 for Windows; ANOVA and Pearson’s correlation analyses were conducted using SPSS 23.0, and all images were plotted using Origin 2021 pro.

## 3 Results

### 3.1 Soil properties between different habitats

As shown in **Supplementary Table S1**, the soil nitrogen and phosphorus contents in the slope habitats were significantly higher than those in the ridge habitats (*P* < 0.05), and the soil pH was less than 7 in both habitats but was lower in the ridge habitats than in the slope habitats. Generally, the soil fertility of the slope habitats was better than that of the ridge.

### 3.2 PFT and RSM variations between different habitats

**Figure 1:**
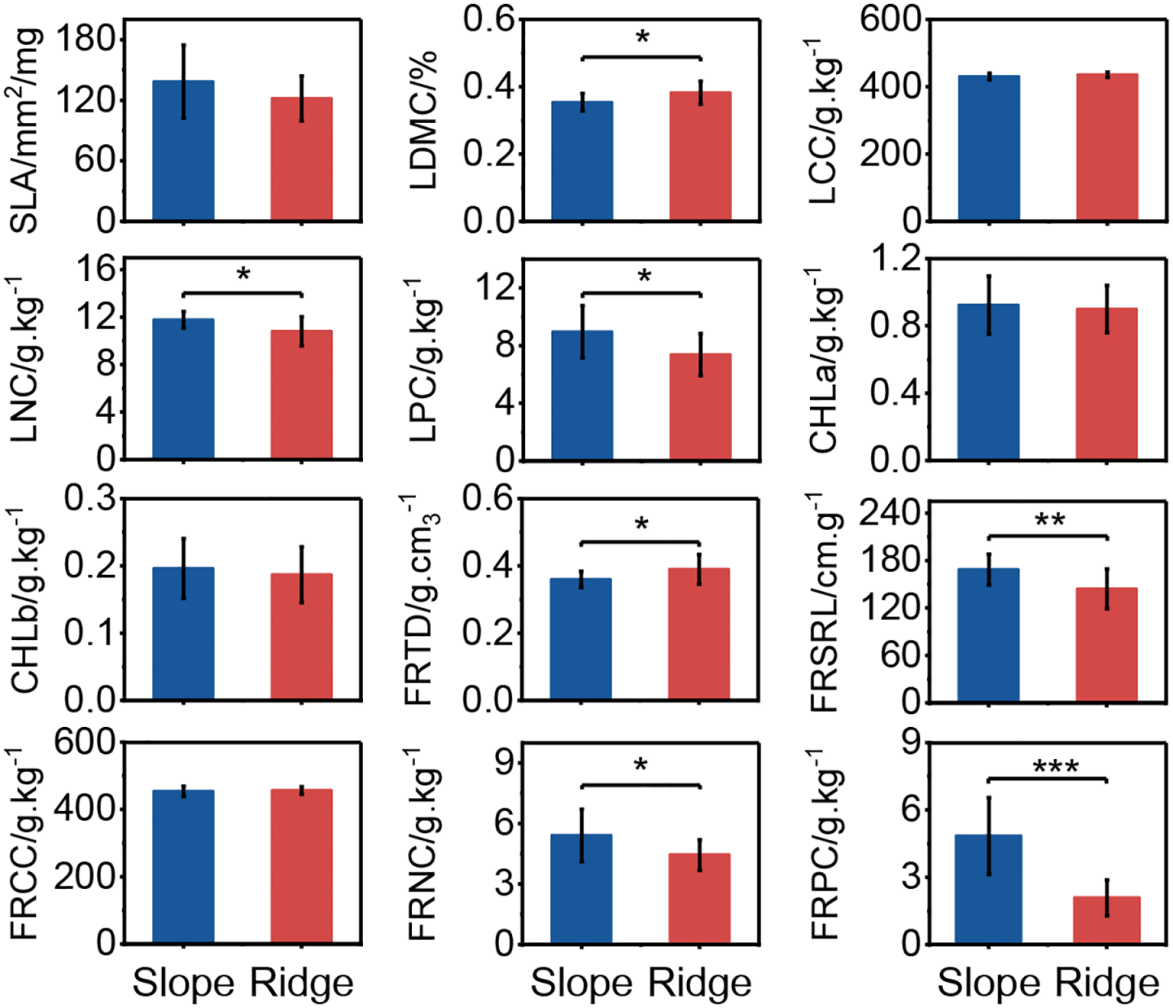
Plant functional trait variations between ridge and slope habitats. Where SLA represents specific leaf area, LDMC represents leaf dry matter content, LCC represents leaf carbon content, LNC represents leaf nitrogen content, LPC represents leaf phosphorus content, CHL represents leaf chlorophyll, FRTD represents fine root tissue density, FRSRL represents fine root specific root length, FRCC represents fine root carbon content, FRNC represents fine root nitrogen content, and FRPC represents fine root phosphorus content. “*” denotes statistically significant differences between groups (*P* < 0.05), “**” denotes statistically very significant differences between groups (*P* < 0.01), same as below. “***” denotes statistically extremely significant differences between groups (*P* < 0.001).

The trait values of the Chinese pine population varied widely between the ridge and slope habitats. LDMC and FRTD were significantly higher in ridge habitats than in slope habitats, whereas LNC, LPC, FRSRL, FRNC, and FRPC were significantly higher in slope habitats than in ridge habitats (*P* < 0.05). The SLA and Chl concentrations in the slope habitats were higher than those in the ridge habitats, but the difference was not significant (**Figure 1**).

**Figure 2:**
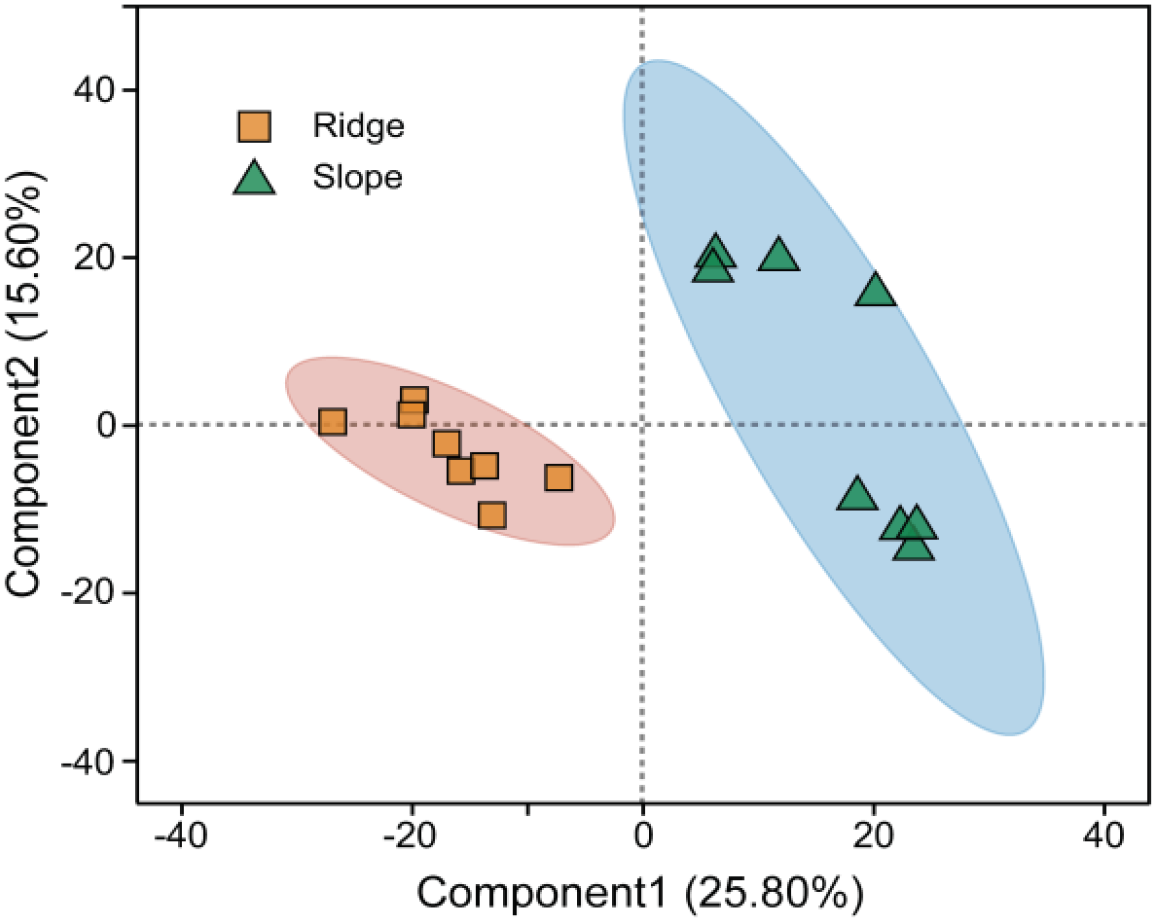
PLS-DA analysis of rhizosphere metabolites in different habitats.

**Figure 3:**
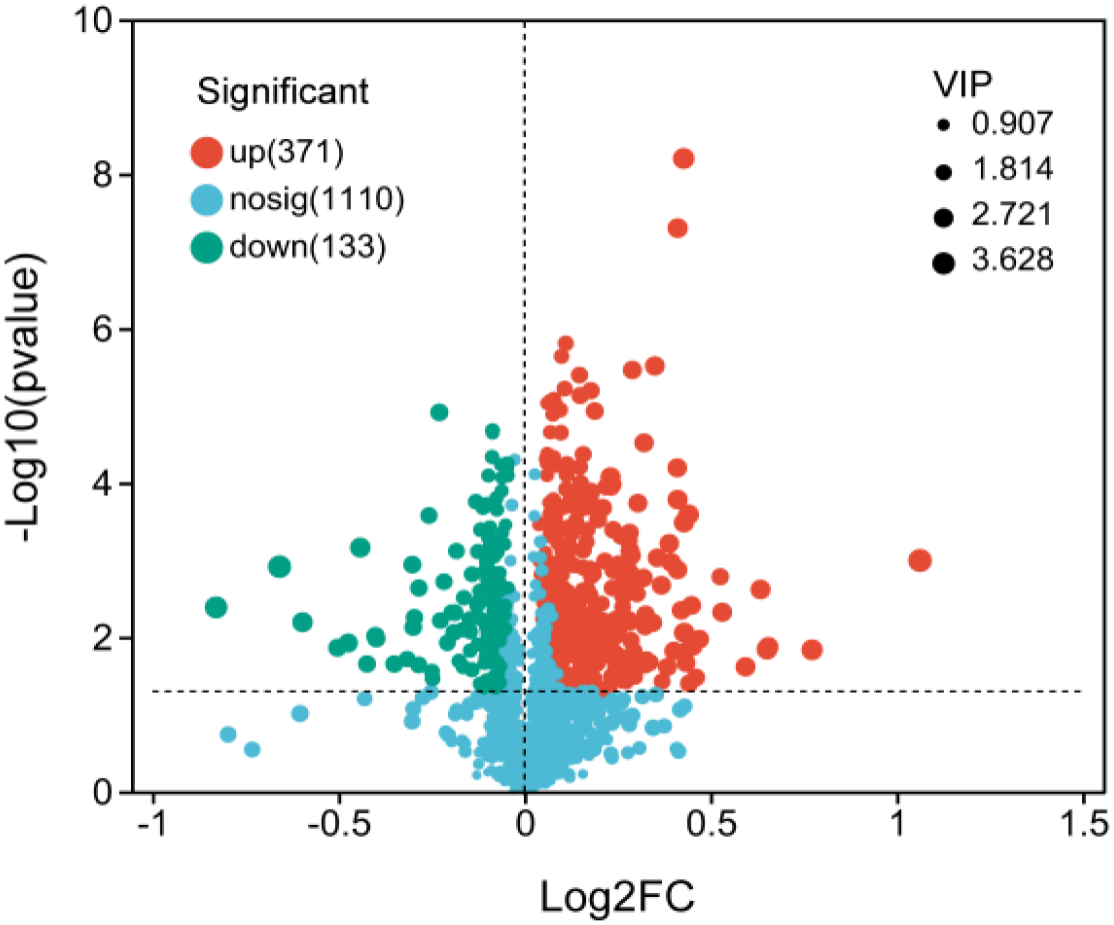
Volcano plots of rhizosphere metabolites for differential habitats.

The metabolite composition varied widely between the two groups (**Figure 2**), and 504 significantly different metabolites were identified. Of these, 377 metabolites were significantly upregulated and 133 metabolites were significantly downregulated in ridge habitats compared to those in slope habitats (**Figure 3**). Among the differential metabolites, the number of lipids and lipid-like molecules, phenylpropanoids and polyketides, organoheterocyclic compounds, and organic oxygen compounds was the highest. In addition to organic acids and their derivatives, the counts of other classes of metabolites in the ridge habitats were significantly higher than those in the slope habitats (**Supplementary Table S2**).

### 3.3 PFT- and RSM-based ecological adaptation strategy variations between different habitats

**Figure 4:**
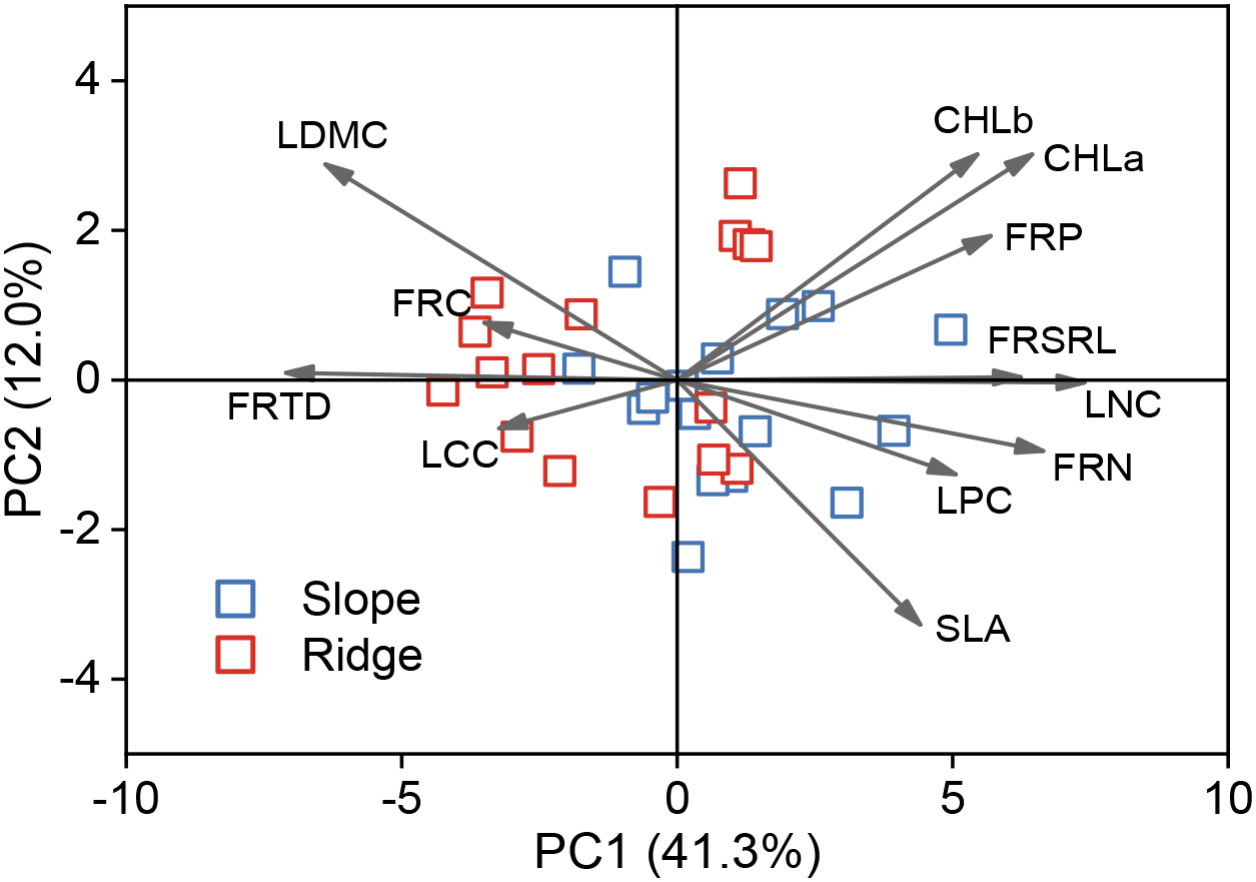
*P.tabuliformis* population functional traits analyzed by principal component analysis (PCA). Points represent plots, and arrows represent the direction of the traits.

PCA was used to analyze the PFT trade-offs. The first PCA axis explained 41.3% of the trait variation and was primarily associated with traits related to safety and efficiency. The population trait values on the left axis show increased strength and durability against barren resources and harsh environments, and the population trait values on the right axis show increased resource acquisition and metabolic efficiency to increase competitive ability (**Figure 4**).

**Figure 5:**
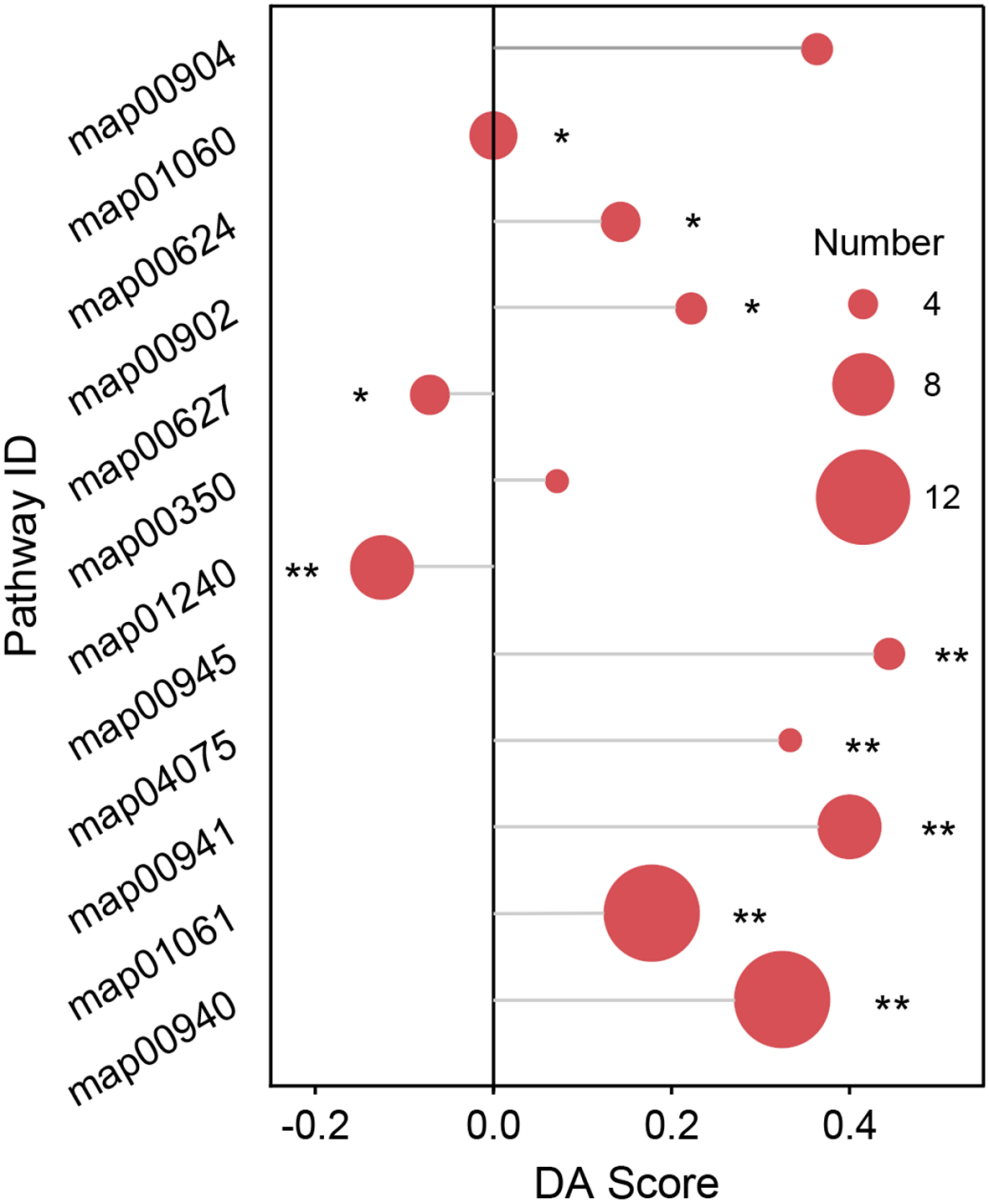
KEGG enrichment analysis of differential metabolites in different habitats. Where map000940 represents phenylpropanoid biosynthesis, map00941 represents flavonoid biosynthesis, map04075 represents plant hormone signal transduction, map00945 represents stilbenoid, diarylheptanoid, and gingerol biosynthesis, map00627 represents aminobenzoate degradation, map00902 represents monoterpenoid biosynthesis, map00624 represents polycyclic aromatic hydrocarbon (PAH) degradation, map01060 represents plant secondary metabolite biosynthesis metabolic pathways. “*” denotes metabolic pathways significant enriched (*P* < 0.05), “**” denotes metabolic pathways highly significant enriched (*P* < 0.01).

We performed metabolic pathway enrichment analysis on the differential metabolites, which showed that: The phenylpropanoid biosynthesis (map00940) and (map01061), flavonoid biosynthesis (map00941), plant hormone signal transduction (map04075), stilbenoid, diarylheptanoid, and gingerol biosynthesis (map00945), aminobenzoate degradation (map00627), monoterpenoid biosynthesis (map00902), polycyclic aromatic hydrocarbon (PAH) degradation (map00624), and plant secondary metabolite biosynthesis (map01060) metabolic pathways were significantly enriched (*P* < 0.05) (**Figure 5**).

**Figure 6:**
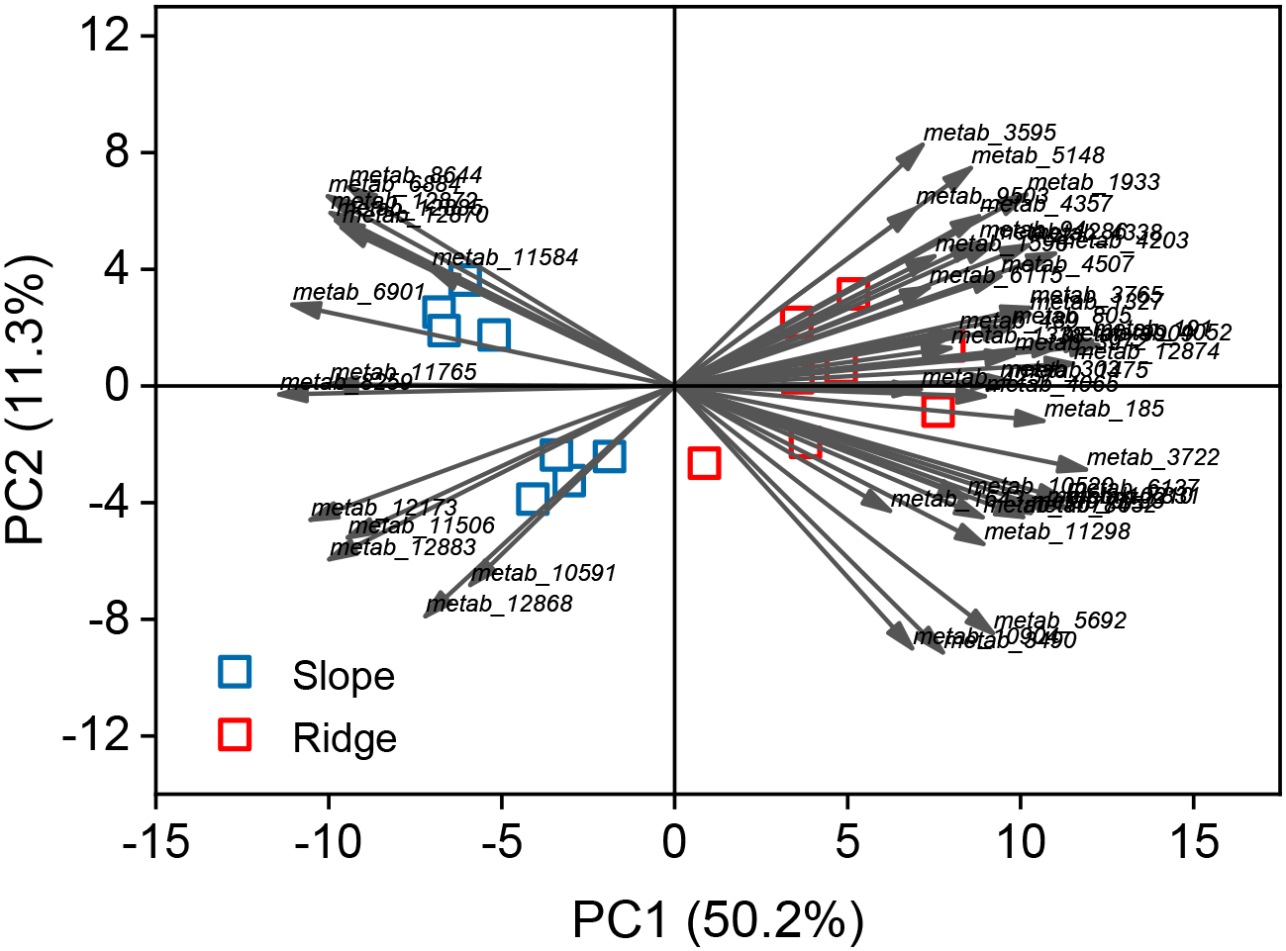
Differential metabolites analyzed by principal component analysis (PCA). Points represent plots, and arrows represent the direction of the differential metabolites.

**Figure 7:**
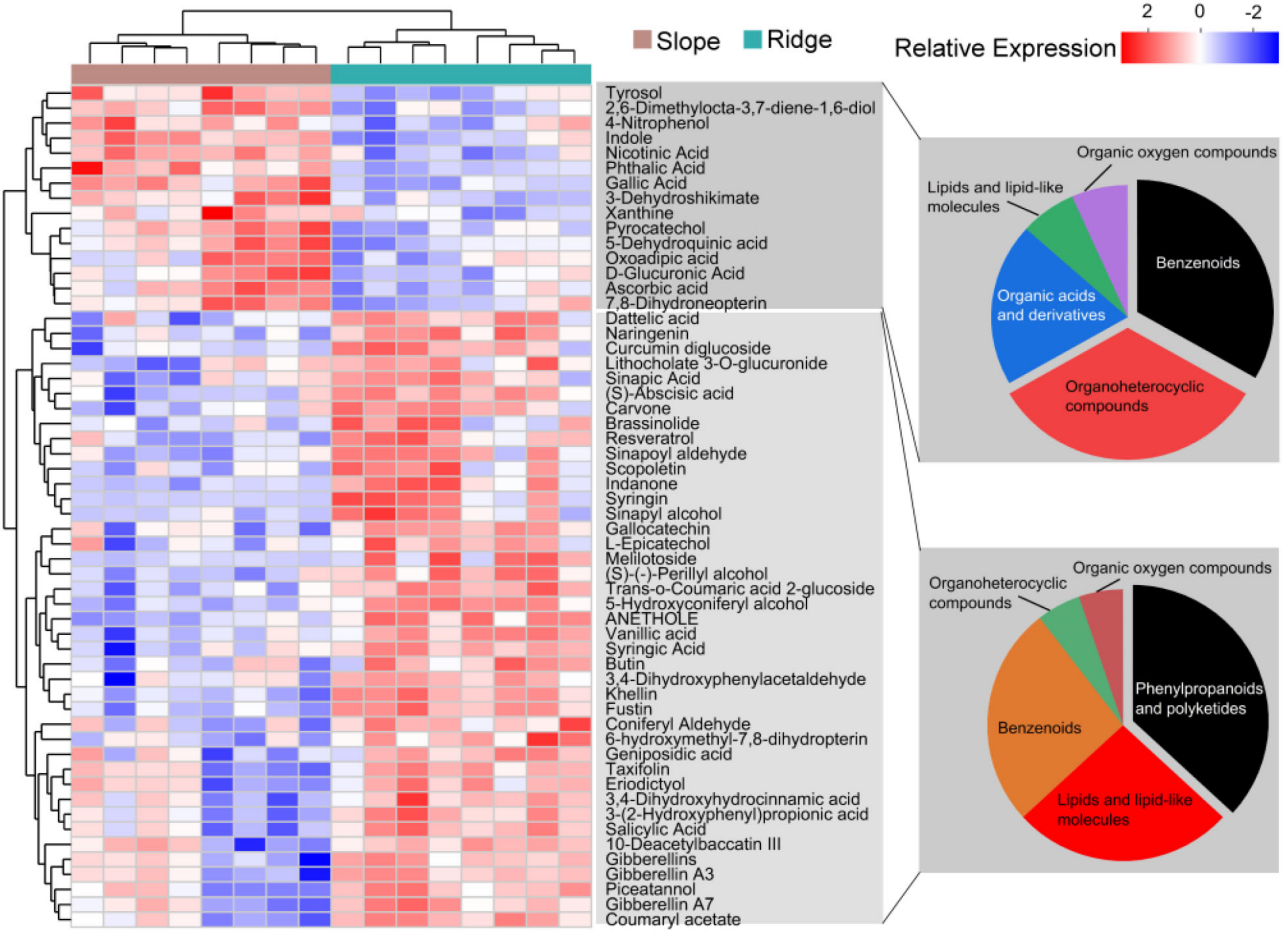
VIP analysis and species composition classification of differential metabolites in the enrichment pathways.

PCA and the composition and classification of metabolites in these metabolic pathways were analyzed. The first PCA axis explained 50.2% of the RSM variation and was reflected with RSMs belonging to up- and down-regulation (**Figure 6**). The metabolites (41) were significantly upregulated in the ridge habitat compared to those in the slope habitat, including phenylpropanoids and polyketides (36.8%), lipids and lipid-like molecules (26.3), and benzenoids (26.3%). Fifteen metabolites were significantly downregulated, including benzenoids (33%), organoheterocyclic compounds (33%), and organic acids and derivatives (20%) (**Figure 7**).

### 3.4 PFT- and RSM-based ecological adaptation strategic associations with environmental factors

**Figure 8:**
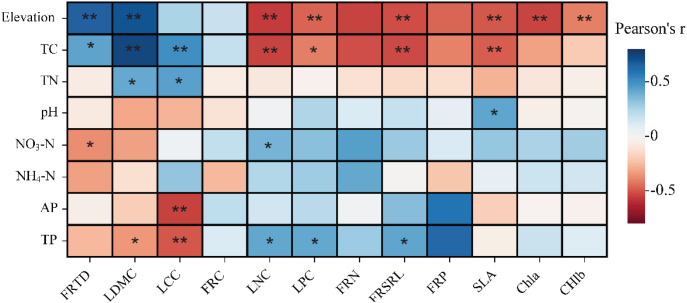
Correlation analysis between traits and environmental factors; “*” represents significant (*P* < 0.05), “**” represents highly significant (*P* < 0.01).

**Figure 9:**
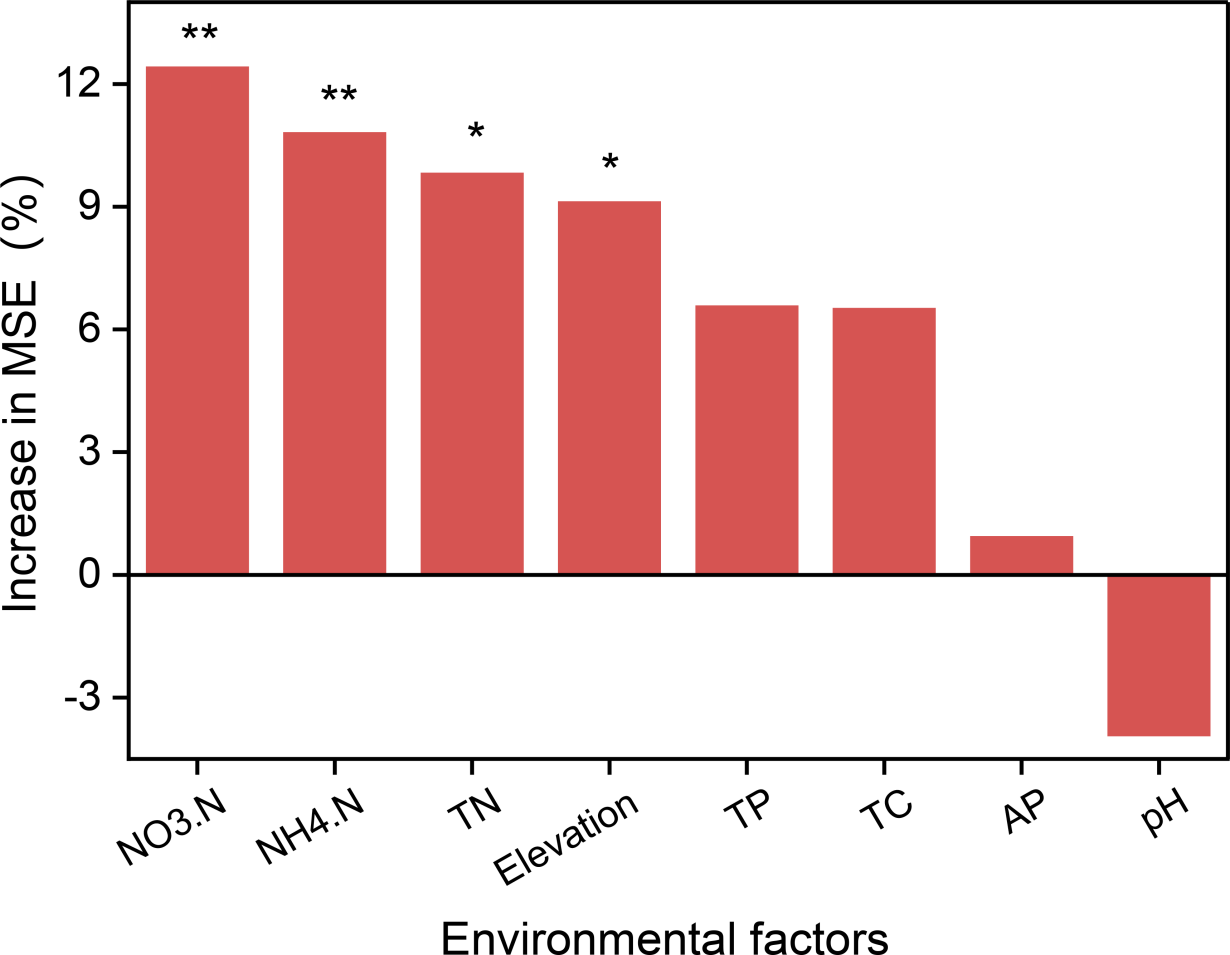
Explanatory degree rank and significance of each factor on the main trait strategy axes. “*” represents significant (*P* < 0.05), “**”represents highly significant (*P* < 0.01).

FRTD, LDMC, and LCC were significantly (*P* < 0.05) negatively correlated with soil nitrogen and phosphorus contents, whereas LNC, LPC, and FRSRL were significantly positively correlated with soil total phosphorus content and significantly negatively correlated with soil carbon content. SLA and Chl content were significantly positively correlated with soil nitrogen and phosphorus content (**Figure 8**). The ranking of environmental factors to explain PFT trade-offs was analyzed using RFM and showed that soil nitrogen and phosphorus contents were the primary factors affecting the trade-offs (**Figure 9**).

**Figure 10:**
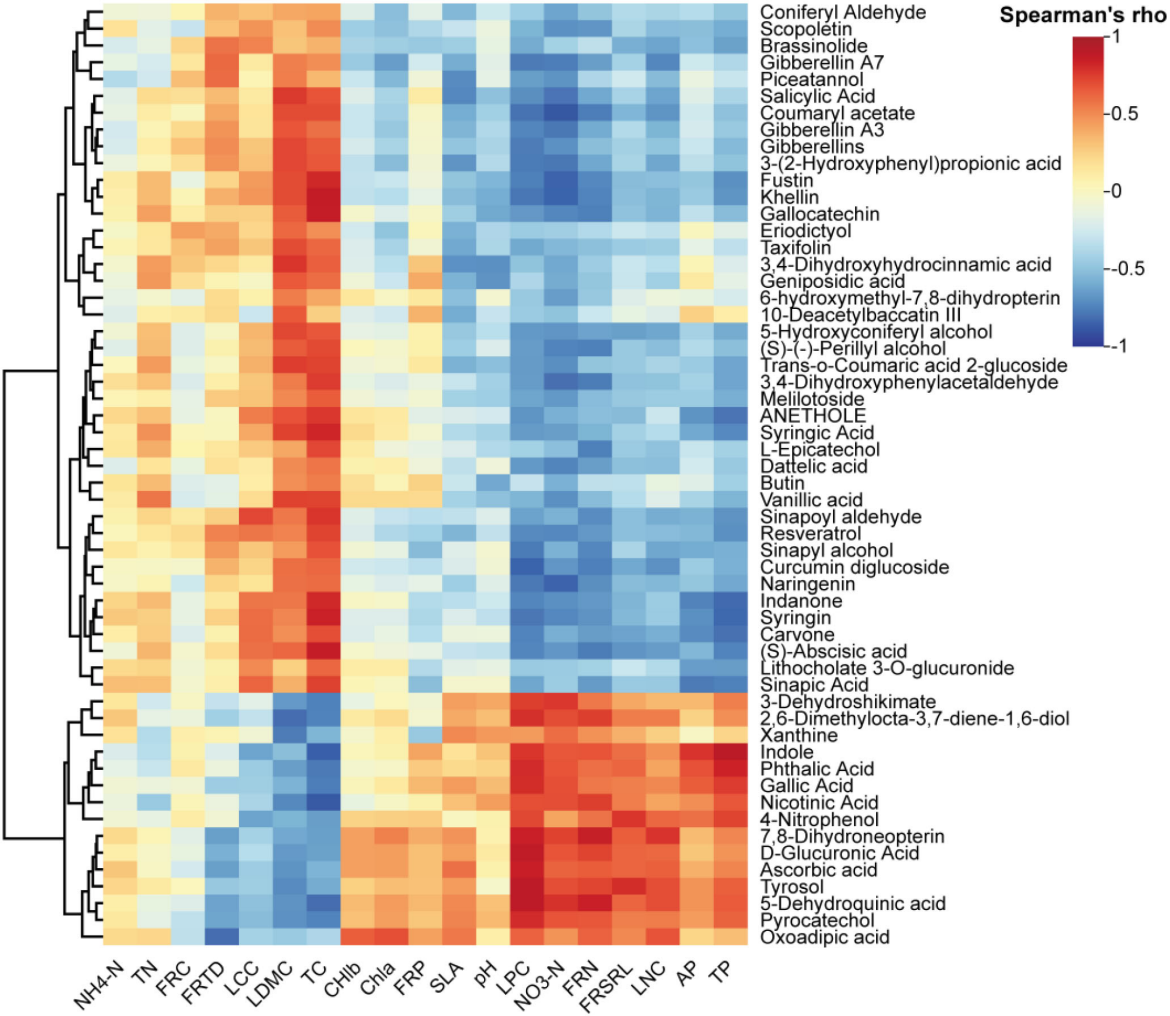
Correlation analysis of the different metabolites with traits and soil factors.

**Figure 11:**
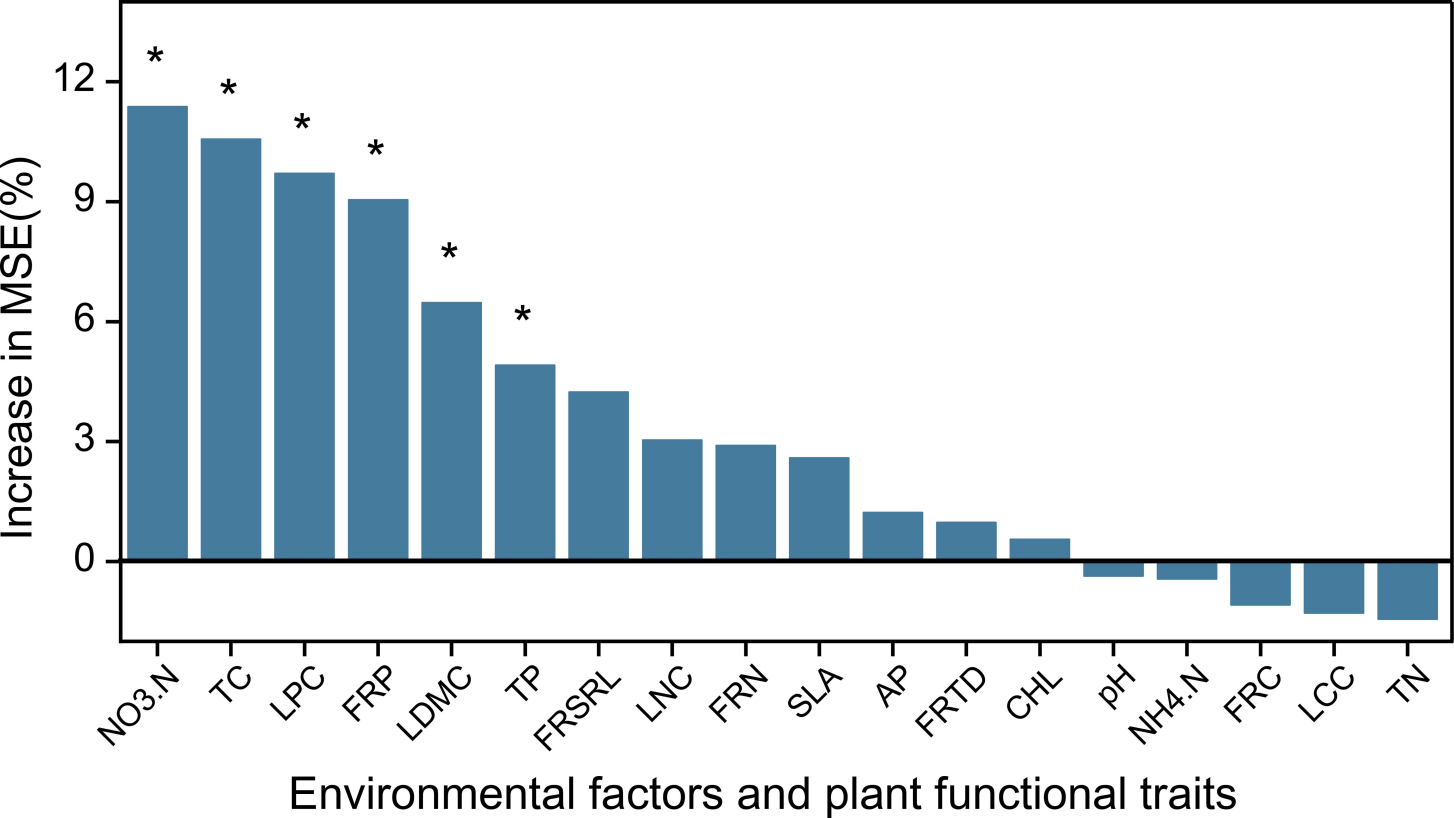
Explanatory degree rank and significance of each factor on differential metabolites. “*” represents significance (*P* < 0.05).

For metabolites in significantly enriched pathways, Spearman’s correlation test revealed distinct correlation patterns with plant traits. Traits associated with resource conservation (e.g., FRC, FRTD, and LDMC) showed significant positive correlations with the upregulated metabolites. In contrast, resource-acquisition traits (such as FRSRL, FRN, FRP, SLA, LNC, LPC, and Chl), along with soil phosphorus and NO_3_^–^N contents, were significantly negatively correlated with these metabolites (**Figure 10**). In addition, the RFM results showed that the regulation of differential rhizosphere metabolites was significantly correlated with NO_3_^–^N, TC, TP, LPC, LDMC and FRP (**Figure 11**).

## 4 Discussion

### 4.1 PFT and RSM variations and trade-offs in different habitats

Within the plant fundamental niche range, owing to the heterogeneity of environmental factors, plants obtain greater habitat adaptability by changing their leaf anatomical, morphological, and physiological traits, as well as trading-off between traits (Zhang, 2017). Our data suggest that SLA and FRSRL in slope habitats were higher than those in ridge habitats, and LDMC and FRTD in ridge habitats were higher than those in slope habitats, which may be due to the fierce competition in slope habitats. Greater SLA has the advantage of intercepting light resources and increasing the photosynthetic rate of plants; ridge habitats have stronger light intensity, smaller LA, and higher LDMC, which will help Chinese pine reduce the loss of needle water by increasing the amount of needle dry matter and the density of mesophyll cells (Holscher et al., 2002; Luo et al., 2005; Poorter et al., 2010). In addition, under relatively tough conditions, plants increase tissue strength, such as the amount of dry matter in needles and RTD, to prolong tissue lifespan and reduce resource waste (Aerts and Chapin, 2000), which is consistent with our results.

Trade-offs are often assumed to be bidirectional, and trait values at the opposite ends of the trade-off axis confer advantages in different environments (Laughlin et al., 2021). As shown in **Figure 2**, the populations on the left of the first axis had high LDMC and FRTD, and low SLA, Chl content, FRSRL, LNC, and LPC, which indicated that populations on the left axis had low photosynthetic capacity and metabolic efficiency, but high tissue strength and durability, and populations on the right showed the opposite traits. The population on the left axis has trait values of increasing strength and durability against barren resources and harsh environments, and the population on the right axis has trait values of increasing resource acquisition and metabolic efficiency to increase competitive ability (Chaturvedi et al., 2011). This functional trait of the *P.tabuliformis* population on the topographical gradient is similar to the results of Maharjan et al.‘s study on species distribution and PFTs on the elevational gradient of the Himalayas, with strategic axes related to plant safety and high metabolic rates to cope with different habitat conditions on an environmental gradient (Maharjan et al., 2021).

### 4.2 Variations and metabolic pathway analysis of RSM in different habitats

Plant RSMs are considered the basis for plant responses to changes in environmental conditions (de Vries et al., 2019; Hamilton and Frank, 2001). In this study, the RSM of *P.tabuliformis* composition varied among different habitats; 377 metabolites significantly upregulated in the ridge habitat compared to the slope habitat (**Supplementary Table S1**). The representative metabolite types included lipids, lipid-like molecules, phenylpropanoids and polyketides. Studies have confirmed that under stress, the accumulation of reactive oxygen species and other substances in plants can induce membrane lipid peroxidation and damage the cell membrane structure and function of the plant. According to the feedback regulation theory, plants prevent, reduce, or repair damage caused by adversity and maintain normal physiological activity through metabolic reactions when exposed to environmental stress. Lipids and lipid-like molecules are not only primary biofilm components but also play an immeasurable role in energy conversion, carbon storage, signal transduction, and stress responses (Yao, 2018), and play an important role in plants, particularly under stress. For example, during salt, drought, and low-temperature stress, phosphatidic acid can accumulate rapidly as a second messenger to regulate various cellular processes, such as stomata, skeleton rearrangement, and vesicle transportation (Zhang and Xiao, 2015; Wang et al., 2018). Furthermore, lipids can regulate the fluidity and stability of cell membranes to protect them from damage or form a lipid-protective membrane on the surface of plant roots to increase cold resistance in plants or prevent harmful microbial colonization (Macabuhay et al., 2021; Zhao et al., 2021). Phenylalanine and trans-cinnamic acid undergo methylation, acylation, and other reactions mediated by different enzymes to produce phenylpropanoids with diverse structures (Lv et al., 2024). Phenylpropanoids are an important class of plant secondary metabolites that exhibit various biological activities, including antibacterial and antioxidant effects. In addition, they have anti-inflammatory, neuroprotective, and immunomodulatory effects in animals and play an important role in biological resistance to biotic and abiotic stresses (Fiorito et al., 2019; Huan et al., 2023).

Adverse stress induces plants to regulate a series of metabolic reactions to defend themselves. Analysis of changes in metabolic pathways during stress is an important method for revealing plant response mechanisms to stress. In the present study, phenylpropanoid biosynthesis, flavonoid biosynthesis, PAH degradation, and other metabolic pathways were significantly enriched. Phenylpropanoid biosynthesis is a secondary plant metabolic pathway primarily used for synthesizing phenylpropanoids. These compounds play important roles in plant growth, development, and defense (Thomas, 2010). Phenylpropanoid biosynthesis is a plant metabolic pathway that begins with phenylalanine and produces various structural polyphenols, including flavonoids, lignin, and coumarin, through a series of biochemical reactions. Flavonoids have antioxidant, antibacterial and antiviral effects, Lignin can enhance the mechanical strength and toughness of plant cells, and coumarin has antibacterial and anti-inflammatory effects, which play an important role in plant responses to environmental changes and stress adaptation (Kim et al., 2020; Yu and Jez, 2010; Wang et al., 2021). Flavonoid biosynthesis involves a series of enzymatic reactions in which phenylalanine is converted into a complex flavonoid structure. Flavonoids are a class of secondary metabolites with various biological functions in plants, including defense against UV-B radiation, resistance to pathogen infection, and promotion of nodule formation and pollen fertility. In addition, flavonoids exhibit a wide range of biological activities, including antioxidant, anti-inflammatory, and antibacterial effects, and play an important role in plant stress resistance and biological adaptability. PAHs are organic compounds that comprise multiple benzene rings (Heitkamp and Cerniglia, 1989). The accumulation of PAHs in plants blocks the stomata of leaves, resulting in leaf discoloration, atrophy, curling, or even shedding. It not only reduces the photosynthetic efficiency and normal physiological functions of plants but also affects their internal environments, causing physiological dysfunction, reducing their resistance to pests and diseases, and leading to plant growth retardation or death (Ji et al., 2023; Peng, 2014). Therefore, PAH degradation enrichment in plants is of great help in improving their stress resistance. The results of the above metabolic pathway analyses were similar to those of previous studies on the metabolism of plants under stress conditions (Bi et al., 2022; Kong et al., 2022), indicating that in ridge habitats, variations in the rhizosphere soil metabolic processes of the *P. tabuliformis* population are an important adaptation strategy for its survival.

### 4.3 Influencing factors of PFT- and RSM-based ecological adaptation strategies

RSMs, including root exudates and rhizosphere soil microorganism metabolism, are involved in plant–microorganism–soil animal interactions and are affected by various biotic and abiotic factors (Hu et al., 2018). Our results illustrate that soil physicochemical properties and PFTs jointly affect the composition of RSMs, with soil NO_3_^–^N, TP, leaf LP, LDMC, and root FRP being the primary influencing factors. NO_3_^–^N and TP concentrations can affect the specific microbial community structure and have an important influence on microbial community succession (Dong et al., 2022). In addition, NO_3_^–^N and TP, as important nutrients, can also affect the growth and development of plants. Leaf phosphate concentration can regulate the catalytic activity of photosynthetic enzymes, and LDMC can regulate the density of mesophyll cells, all of which have important effects on the photosynthetic efficiency of plants (Fleisher et al., 2012). Therefore, plants and rhizosphere microbial communities develop different traits and metabolites according to external environmental conditions to adapt to resource deficiency or environmental stress.

A combined analysis of PFT trade-offs and RSM pathways showed that *P.tabuliformis* populations in ridge and slope habitats employ distinct ecological adaptation strategies. Interestingly, the conservative strategy inferred from plant functional trait tradeoffs was aligned with the metabolic profile characterized by the upregulation of key metabolites in enriched pathways. This consistent pattern is supported by the strong correlation between specific traits and specific metabolites and the shared response of both traits and metabolites to the same key environmental driver, as revealed by the random forest model. Overall, in the slope habitat with better soil conditions, the *P.tabuliformis* population adopted a resource acquisition strategy to obtain greater resource competitiveness and resource utilization efficiency. In the ridge habitat, to adapt to harsh resource conditions, such as low soil fertility, the *P. tabuliformis* population adopted a resource-conservation strategy to reduce nitrogen and phosphorus concentrations in needles and roots, enhance their tissue density and strength to prolong the life of needles and fine roots, and reduce the metabolic rate of organisms.

## 5 Conclusions

Populations in ridge and slope habitats have shown major strategy adaptations from resource conservation to resource acquisition. The secretion pattern of RSMs was also strategic among different habitats, and the relative contents of defense metabolites, such as ketones, lipids, and phenols, were significantly upregulated with the deterioration of habitat conditions, and the metabolite profile is functionally aligned with the ecological strategy. The PFT-based strategies were consistent, and soil nutrients were the primary factors affecting the ecological adaptation strategies of *P.tabuliormis* populations in different habitats. However, it is important to acknowledge that other unmeasured environmental variables, such as light availability and temperature fluctuations inherent to the ridge and slope habitats, may have also contributed to the observed strategic adaptations and warrant further investigation.

## Data Availability

The original contributions presented in the study are included in the article/**Supplementary Material**, further inquiries can be directed to the corresponding author/s.
