# Supplementary material for: Habitat-driven ecological strategies shape Chinese pine functional traits and rhizosphere metabolites in Qinling Mountains, China
Source: Front Plant Sci. 2025 Nov 18;16:1690544. doi: 10.3389/fpls.2025.1690544 (PMC12668977; doi:10.3389/fpls.2025.1690544)
Supplement: Supplementary file 1 [file Table1.docx]

**Table.S1** Plant diversity and soil physicochemical properties in different forest types.

| Parameters | Slope | Ridge |
| --- | --- | --- |
| pH | 5.50±0.08 a | 5.02±0.07 b |
| SOC | 14.53±1.74 b | 19.33±1.27 a |
| TN | 1.89±0.14 a | 1.51±0.08 b |
| TP | 1.23±0.24 a | 0.5±0.1 b |
| AP | 0.36±0.49 a | 0.13±0.18 a |
| NH4^+^-N | 13.93±4.06 a | 11.24±1.66 a |
| NO3^-^-N | 3.64±1.94 a | 3.34±2.15 a |

pH: Pondus hydrogenii, SOC: Soil organic carbon, TN: Soil total nitrogen, TP: Soil total phosphorous, AP: Soil available phosphorus, NH_4_^+^-N: Soil ammonium N, NO_3_^-^-N: Soil nitrate N. Different lowercase letters indicate that the average values of soil physicochemical properties and plant diversity index at different forest ages under the same forest type differ significantly (*p* <0.05).

**Table S2** Quantitative characteristics of differential metabolites between different habitats

| Metabolite class | Ridge vs. Slope | | Total |
| --- | --- | --- | --- |
|  | Up regulated | Down regulated |  |
| Lipids and lipid-like molecules | 116 | 20 | 136 |
| Phenylpropanoids and polyketides | 74 | 11 | 85 |
| Organoheterocyclic compounds | 43 | 25 | 68 |
| Organic oxygen compounds | 46 | 15 | 61 |
| Organic acids and derivatives | 20 | 30 | 50 |
| Benzenoids | 31 | 8 | 39 |
| Lignans, neolignans and related compounds | 4 | 0 | 4 |
| Nucleosides, nucleotides, and analogues | 2 | 2 | 4 |
| Alkaloids and derivatives | 3 | 0 | 3 |
| Organic nitrogen compounds | 2 | 1 | 3 |
| Not Available | 1 | 1 | 2 |
| Hydrocarbons | 1 | 0 | 1 |
